# Low Molecular Weight Collagen Peptide (LMWCP) Promotes Hair Growth by Activating the Wnt/GSK-3β/β-Catenin Signaling Pathway

**DOI:** 10.4014/jmb.2308.08013

**Published:** 2023-09-30

**Authors:** Yujin Kim, Jung Ok Lee, Jung Min Lee, Mun-Hoe Lee, Hyeong-Min Kim, Hee-Chul Chung, Do-Un Kim, Jin-Hee Lee, Beom Joon Kim

**Affiliations:** 1 Department of Dermatology, College of Medicine, Chung-Ang University, Seoul 06974, Republic of Korea; 2 Department of Medicine, Graduate School, Chung-Ang University, Seoul 06973, Republic of Korea; 3 Health Food Research and Development, NEWTREE Co., Ltd., Seoul 05604, Republic of Korea

**Keywords:** LMWCP (low molecular weight collagen peptide), β-catenin, human dermal papilla cells (hDPCs), VEGF, Wnt3a

## Abstract

Low molecular weight collagen peptide (LMWCP) is a collagen hydrolysate derived from fish. We investigated the effects of LMWCP on hair growth using human dermal papilla cells (hDPCs), human hair follicles (hHFs), patch assay, and telogenic C57BL/6 mice, while also examining the underlying mechanisms of its action. LMWCP promoted proliferation and mitochondrial potential, and the secretion of hair growth-related factors, such as EGF, HB-EGF, FGF-4, and FGF-6 in hDPCs. Patch assay showed that LMWCP increased the neogeneration of new HFs in a dose-dependent manner. This result correlated with an increase in the expression of dermal papilla (DP) signature genes such as, *ALPL*, *SHH*, *FGF7*, and *BMP-2*. LMWCP upregulated phosphorylation of glycogen synthase kinase-3β (GSK-3β) and β-catenin, and nuclear translocation of β-catenin, and it increased the expression of Wnt3a, LEF1, VEGF, ALP, and β-catenin. LMWCP promoted the growth of hHFs and increased the expression of β-catenin and VEGF. Oral administration of LMWCP to mice significantly stimulated hair growth. The expression of Wnt3a, β-catenin, PCNA, Cyclin D1, and VEGF was also elevated in the back skin of the mice. Furthermore, LMWCP increased the expression of cytokeratin and Keratin Type I and II. Collectively, these findings demonstrate that LMWCP has the potential to increase hair growth via activating the Wnt/β-catenin signaling pathway.

## Introduction

Alopecia is on the rise regardless of gender and age. Currently, there are two drugs approved by the United States Food and Drug Administration (US FDA): Finasteride and Minoxidil (MNX)[1]. Finasteride promotes a decrease in the concentration of dihydrotestosterone (DHT), which induces apoptosis in human dermal papilla cells (hDPCs) [1]. MNX enhances the nutrient supply to hair follicles through vasodilation [2]. However, these drugs have side effects such as allergic contact dermatitis and itching. Furthermore, discontinuation of MNX leads to the recurrence of alopecia, while prolonged use of finasteride can cause sexual dysfunction [3, 4]. Therefore, there is a significant demand for new hair loss treatment options with fewer side effects and easier accessibility.

Human hair follicles (hHFs) are composed of epidermal (epithelial) and dermal (mesenchymal) compartments, and their communication is crucial in the morphogenesis and growth of HFs [5, 6]. hDPCs play a pivotal role in the regulation of growth, formation, and cycling of hHF mainly through reciprocal interactions with surrounding epithelial cells [7, 8]. Growth factors, including insulin-like growth factor-1 (IGF-1), hepatocyte growth factor (HGF), vascular endothelial growth factor (VEGF), and keratinocyte growth factor (KGF), secreted from hDPCs stimulate keratinocytes to proliferate and differentiate into the hair shaft during the anagen phase [9, 10].

Wnt/β-catenin signaling pathway is important in regulating the growth cycle of hair follicles [11, 12]. Its activation promotes the proliferation and migration of hair follicle stem-cells, hair matrix cells, and hDPCs [13, 14], thereby inducing the transition of hHFs from telogen to anagen. In addition, this pathway can promote angiogenesis and provide a nutrient-rich environment for hHFs growth [15, 16]. Therefore, targeting this pathway represents a potential approach for hair-loss prevention and treatment.

hHFs undergo repetitive cycles of growth (anagen), regression (catagen), and rest (telogen) [17]. The volume of dermal papilla (DP) in hHFs is greatly affected by the amount of collagen during the anagen phase. Collagen is essential for increasing hair thickness [18], making collagen an important factor in maintaining normal hair growth. However, skin aging promotes the cleavage of collagen through the activation of matrix metalloproteinases (MMPs), and this collagen loss is not completely replenished by *de novo* collagen synthesis [19]. Therefore, supplementing external collagen can help maintain healthy hair during aging.

The low molecular weight collagen peptide (LMWCP) is derived from a skin of pangasius hypophthalmus, and a collagen hydrolysate containing 3% Gly-Pro-Hyp and 15% tripeptide [20]. According to the previous studies, LMWCP has various health benefits, including wrinkle reduction, increased hydration and elasticity, and cartilage regeneration [21, 22]. Interestingly, Hyunju *et al*. revealed that collagen plays pivotal roles in spheroid formation and anti-aging of hDPCs [18]. Furthermore, Christian *et al*. reported that collagen crosslinking due to photoaging in the scalp increases with age, impacting the remodeling of the hHFs during early anagen as it moves downwards in the dermis [23]. These results indicate that collagen is a key regulator for hair growth and hair health.

Considering the above data, LMWCP, which is mainly composed of collagen, can play a positive role in regulating and maintaining hair growth. Thus, in this study, we investigated the effect of LMWCP on hair growth and explored the mechanism underlying its hair growth-promoting activity using the anagen induction assay in telogenic C57BL/6 mice, as well as patch assays, hHF organ cultures, and hDPCs.

## Materials and Methods

### LMWCP and Dermal Papilla Cell Culture

LMWCP supplied by NEWTREE Co., Ltd., South Korea was prepared by spray drying the gelatin hydrolysate obtained by enzymatic degradation of gelatin derived from a skin of pangasius hypophthalmus using a protease and was standardized based on Gly-Pro-Hyp (3%) and tripeptide (≥15%) contents. hDPCs were purchased from PromoCell (Germany). The cells were maintained in an incubator with 5% CO_2_ at 37°C using a follicle dermal papilla cell growth medium kit (PromoCell) and subcultured upon reaching 70-80% confluency.

### Cell Viability Assay

hDPCs were seeded in 96-well plates and cultured to reach a confluency of 90%. After 24 h, the cells were treated with 0, 0.1, 0.3, 1, and 3 mg/ml of LMWCP for 24 h. Cell viability was quantified using a WST-8 assay kit (QuantiMax, Biomax, Korea). Absorbance was measured at 450 nm using a microplate spectrophotometer (SpectraMax 340; Molecular Devices, Inc., USA).

### Mitochondrial Bioenergetics Assessment

Mitochondrial membrane potential was measured using a JC-1 mitochondrial membrane potential assay kit (Abcam, UK). Briefly, hDPCs treated with LMWCP or MNX (Sigma-Aldrich, USA) were stained with 1 μM JC-1 solution. Fluorescence intensities from JC-1 aggregate and monomer forms were measured at 590 nm (535 nm excitation) and 530 nm (475 nm excitation), respectively, using a microplate spectrophotometer (SpectraMax 340; Molecular Devices, Inc., USA). Mitochondrial membrane potential (ΔΨm) was visualized by capturing fluorescence images using a fluorescence microscope (DMi8, Leica, Germany).

### Immuno Blot Assay

Total cellular proteins from hDPCs were collected and lysed in RIPA buffer (Thermo Fisher Scientific, USA). Protein samples (30 μg) were analyzed using western western blot assay with the following antibodies; Cytokeratin antibody (Sigma-Aldrich); VEGF and β-actin antibody (Santa Cruz Biotechnology, USA); Alkaline phosphatase (ALP) antibody (Thermo Fisher Scientific); Cyclin D1, p-GSK-3β (Ser^9^), GSK-3β, p-β-catenin (Ser^675^), p-β-catenin (Ser^33/37^/Thr^41^), total β-catenin, p-PKA(Thr^197^), PKA, LEF1, CDK2, p-AKT(Ser^473^), AKT, β-actin, and proliferating cell nuclear antigen (PCNA) antibody (Cell Signaling Technology Inc. USA); type I + II hair keratin antibody (Progen Biotechnical GmbH, Germany); Cyclin E, CDK6, and Wnt3a antibody (Abcam, UK). Protein bands were visualized using a ChemiDoc MP Imaging System (Bio-Rad Laboratories, Inc., USA). The resulting blots were analyzed using NIH Image J software (Bethesda, USA).

### Immunocytochemistry (ICC)

The cells were fixed with 4% paraformaldehyde (PFA) for 30 min, washed with PBS (phosphate-buffered saline), blocked with 3% BSA (bovine serum albumin) and 0.2% Triton X-100 in PBS at room temperature (RT) for 1 h, and incubated with primary antibodies overnight at 4°C. After washing with PBS, the cells were incubated with anti-rabbit IgG-FITC secondary antibodies (Santa Cruz Biotechnology) in the dark at RT for 1 h. The cell nuclei were counter-stained with 4',6-Diamidino-2-Phenylindole, dihydrochloride (DAPI) (Immuno Bioscience Corp., USA), and the cells were observed using confocal microscopy (LSM 880, Zeiss, Germany).

### Quantitative RT-PCR (qRT-PCR) Analysis

Total RNA was extracted using the TRIzol reagent (Invitrogen, USA). cDNA synthesis was performed using Prime Script TM RT Master Mix (Takara, Japan). Quantitative PCR was performed using qPCR 2X PreMIX SYBR (Enzynomics, Korea) on a CFX96 Touch Real-Time PCR Detection System (Bio-Rad). Gene expression levels were calculated and reported as cycle threshold (Ct) values using the ΔCt quantification method. Glyceraldehyde-3-phosphate dehydrogenase (*GAPDH*) was used for normalization. The primers used for qPCR are summarized in Table 1.

### Growth Factor Antibody Array

A human growth factor antibody array membrane kit (Abcam) was used to measure changes in the profiles of the growth factors in hDPCs following LMWCP treatment. Briefly, hDPCs (5 × 10^5^ cells/well) were seeded in 6-well plates and cultured overnight. Cells were treated with 3 mg/ml of LMWCP for 24 h, and the culture supernatants were collected for growth factor analysis. Fresh medium and culture supernatant from non-treated cells were used as blank and control, respectively. The resulting blots were analyzed under identical conditions using a chemiluminescence EZ-capture (Atto, USA).

### Human HF Organ Culture

All hair follicles were obtained from Dankook University Hospital (ethical approval number 2019M-008). The isolated anagen follicles were cultured in 500 μl of Williams E medium (Gibco, USA) at 37°C with 5% CO_2_. After 24 h, the hHFs were cultured with 1 or 3 mg/ml LMWCP or MNX (50 μM) for 8 days. The pictures of the hair follicles were obtained using a stereo microscope (Zeiss). On day 8, each hHF was evaluated as either in anagen VI (score 1), early catagen (score 2), mid-catagen (score 3), or late catagen (score 4), and the anagen/catagen ratio was calculated for each group. hHFs elongation was analyzed using ImageJ (version 1.52a). The hHFs were then fixed in 10% formalin.

### Histology and Immunohistochemistry (IHC)

Dorsal skin tissues from each mouse were fixed with 10% formalin, embedded in paraffin, and then cut into sections that were stained with hematoxylin and eosin (H&E). For IHC, the sliced sections were incubated with primary antibodies. The stained slides were photographed using a slide scanner (Pannoramic MIDI; 3DHISTECH Ltd, Hungary) and observed using Case Viewer software. The number of hHFs was counted on a cropped image in a fixed area (1 × 1 mm).

### Patch Assay

Truncal skin was removed from newborn C57BL/6 mice and rinsed in Dulbeccós phosphate-buffered saline (DPBS). The skin was washed with a povidone-iodine solution and incubated with Collagenase/Dispase (2.5 mg/ml; Roche, Switzerland) overnight at 4°C. Afterward, dermal cells and epidermal cells were isolated, and 0.25%trypsin-EDTA was added to each cell population, followed by incubation at 37°C for 2 h. The cells were centrifuged at 2,000 rpm for 20 min at 4°C. A cell mixture of 1 × 10^6^ dermal cells and 5 × 10^5^ epidermal cells were re-suspended in DMEM-F12 medium (Hyclone) and injected (26-gauge needle) into the hypodermis of BALB/c nude mice. The dorsal skin of the mice was monitored and photographed using a digital camera for 14 days.

### Hair Regeneration Model

C57BL/6 mice (six-week-old, male) were purchased from Saeron Bio Inc. (Korea) and acclimated for 1 week. The mice were maintained at 23 ± 2°C and 50 ± 10% humidity with a 12-h light/12-h dark cycle. All animal experiments were conducted according to the Principles of Laboratory Animal Care established by the National Institutes of Health (NIH) and were approved by the Chung-Ang University Institutional Animal Care and Use Committee (IACUC No. A2022018). The mice were randomly divided into four groups: normal control (*n* = 7), LMWCP 615 mg/kg (*n* = 7), LMWCP 820 mg/kg (*n* = 7), and MNX 3% (*n* = 7). LMWCP was administered once a day for two weeks through oral administration, while MNX was administered topically. To compare the growth rate, the dorsal skin was photographed using a digital camera on days 0, 10, and 13 after depilation using hair removal cream, and the ratio growth area/total area was calculated using ImageJ software. The dorsal skin was removed for histological analysis after euthanizing the mice on days 0, 10, and 13.

### Statistical Analysis

All data are reported as the mean ± standard deviation (SD) of at least three independent experiments. The data were analyzed using one-way analysis of variance (ANOVA) followed by a Bonferroni post hoc test. All statistical analyses were performed using the GraphPad Prism 7.0 software (GraphPad Software Inc., USA). Differences with p values lower than 0.05 were considered statistically significant and indicated with the following symbols: *, *p* < 0.05; **, *p* < 0.01; ***, *p* < 0.001; and ****, *p* < 0.0001.

## Results

### LMWCP Increases Proliferation and Mitochondrial Potential of hDPCs

**Fig. 1:**
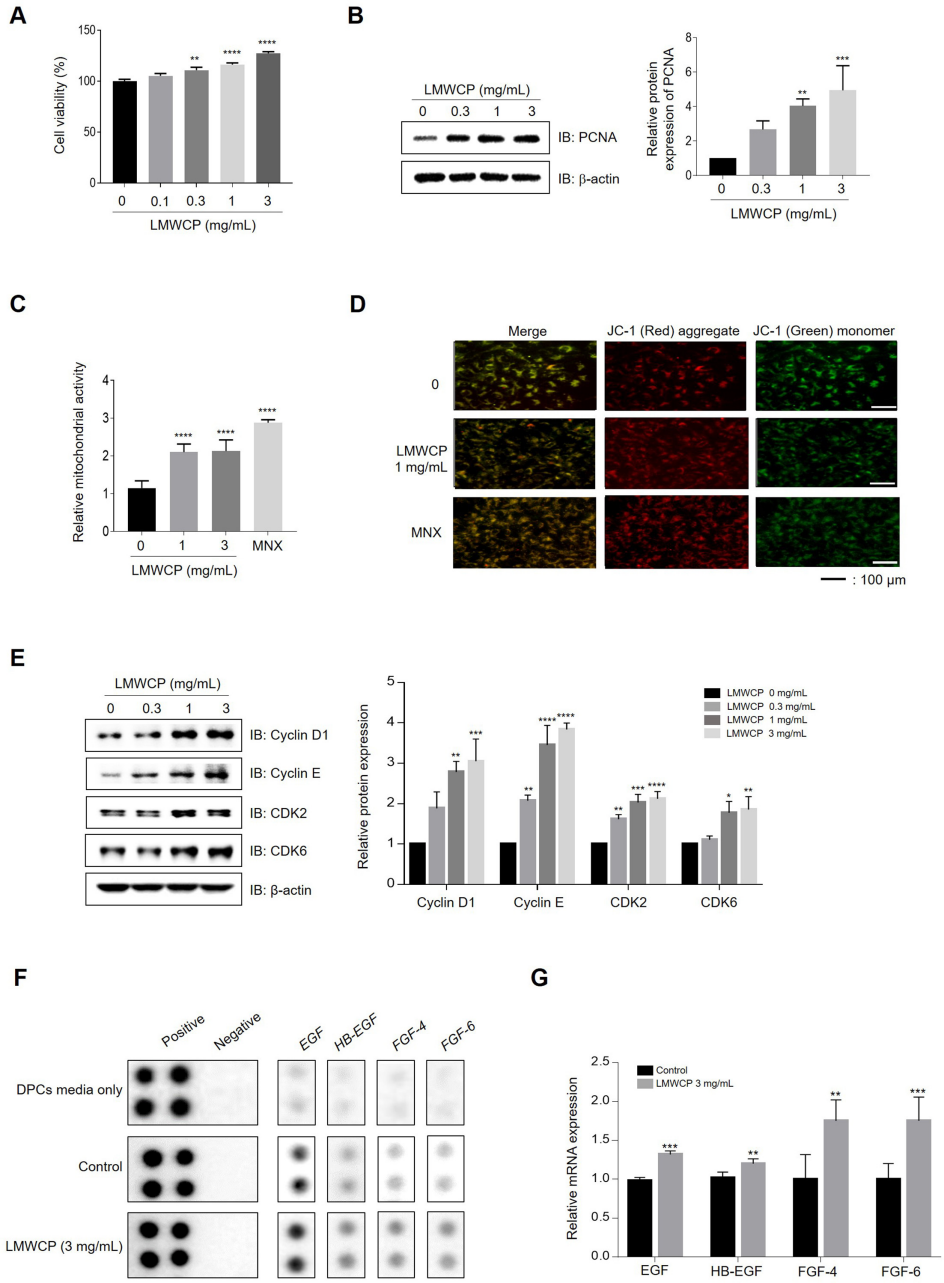
Effect of the LMWCP on proliferation and cellular energy metabolism in hDPCs. (**A**) Proliferation of hDPCs was assessed after LMWCP treatment (0, 0.1, 0.3, 1, and 3 mg/ml) for 24 h. (**B**) The expression of PCNA after treatment with a LMWCP for 24 h. (**C**) JC-1 aggregates (A590)/monomer (A530) ratio of DPCs treated with LMWCP (0, 1, and 3 mg/ml) and MNX (1 μM) for 24 h. (**D**) JC-1 monomer form (green) and aggregate form (red) were detected using fluorescent microscopy. (**E**) The expression of cyclin D1, cyclin E, CDK2, and CDK6 after treatment with LMWCP for 24 h. (**E**) Cultured media from hDPCs treated with either vehicle, growth media, or LMWCP (0, 0.3, 1, and 3 mg/ml) for 48 h was used for analysis using the growth factor antibody array. (**F**) The mRNA expression levels of *EGF, HB-EGF, FGF-4*, and *FGF-6* in hDPCs treated with LMWCP (3 mg/ml) for 1 h were analyzed by qPCR (*n* = 3). The results are shown as the mean ± standard deviation. *, *p* < 0.05; **, *p* < 0.01; ***, *p* < 0.001; ****, *p* < 0.0001 compared to the control group.

LMWCP significantly increased the proliferation of hDPCs by 10–30% at concentrations of 0, 0.1, 0.3, 1, and 3 mg/ml (Fig. 1A), and it also upregulated the expression of PCNA (proliferating cell nuclear antigen), a cellular marker for proliferation (Fig. 1B). Mitochondrial β-oxidation favorably impacts hair growth in vitro [24, 25]. Mitochondrial membrane potential increased by 83% and 85% upon LMWCP treatment at concentrations of 1 and 3mg/ml, respectively (Fig. 1C). Highly activated mitochondrial membrane potential was visualized using fluorescence microscopy, where red dots were enhanced while green dots were reduced by LMWCP in cultured hDPCs (Fig. 1D). LMWCP also increased the protein levels of cyclin D1, cyclin E, cyclin-dependent kinase 2 (CDK2), and cyclin-dependent kinase 6 (CDK6) in a dose-dependent manner (Fig. 1E). To investigate the effect of LMWCP on hair growth factor secretion in hDPCs, we performed a human growth factor antibody array analysis. LMWCP significantly increased the secretion of EGF, HB-EGF, FGF-4, and FGF-6, which are known to stimulate hair growth (Fig. 1F) [17]. Additionally, using qRT-PCR, we confirmed that the expression levels of these factors were significantly increased in LMWCP-treated hDPCs (Fig. 1G).

### LMWCP Activates Wnt /GSK-3β/β-Catenin Signaling Pathway

**Fig. 2:**
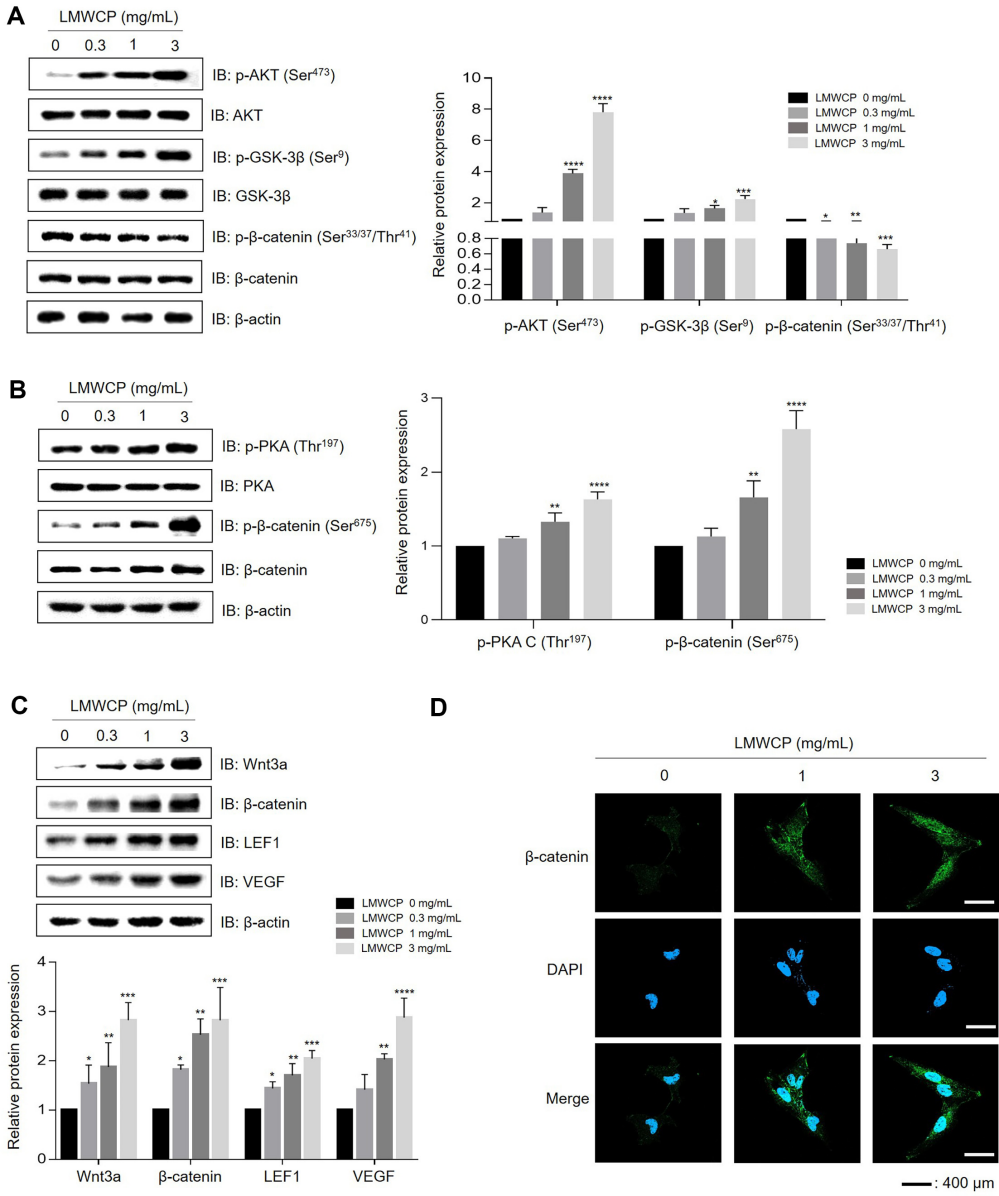
Effect of LMWCP on the Wnt-AKT-GSK-3β/β-catenin pathway. (**A**) hDPCs treated with LMWCP (0, 0.3, 1, and 3 mg/ml) for 1 h were lysed and analyzed for p-AKT(ser^473^), AKT, p-GSK(Ser^9^), GSK, p-β-catenin(Ser^675^), p-β-catenin (Ser^33/37^/Thr^41^), β-catenin, PKA, p-PKA (Thr ^197^), and β-actin. (**B**) hDPCs treated with a LMWCP (0, 0.3, 1, and 3 mg/ml) for 24 h were analyzed for Wnt3a, LEF1, β-catenin, VEGF, and β-actin. (**C**) Expression of β-catenin was analyzed by ICC. Representative data from three independent experiments are shown. The results are shown as the mean ± standard deviation. *, *p* < 0.05; **, *p* < 0.01; ***, *p* < 0.001; ****, *p* < 0.0001 compared to the control group.

GSK-3β/β-catenin signaling is necessary for the regulation of diverse biological events, including cell proliferation, hair growth, and hair regeneration [11, 12]. Upon LMWCP treatment, the levels of p-Akt (Ser473) and the phosphorylation of glycogen synthase kinase-3b (GSK-3b) on Ser^9^ increased in a dose-dependent manner. One other hands, LMWCP decreased the phosphorylation of β-catenin on Ser^33/37^/Thr^41^(Fig. 2A). At the same time, LMWCP treatment also increased the phosphorylation of PKA on Thr^197^ and β-catenin on Ser^675^ (Fig. 2B), leading to less proteosomal degradation and more nuclear translocation and activation of β-catenin. We confirmed that LMWCP increased the expression levels of wnt family member 3a (Wnt3a), β-catenin, lymphoid Enhancer Binding Factor 1(LEF1), and vascular endothelial growth factor (VEGF) in a dose-dependent manner (Fig. 2C). By ICC, we observed that the increased translocation of β-catenin to the nucleus in LMWCP-treated hDPCs compared to controls (Fig. 2D). These results indicate that LMWCP activates the Wnt-AKT-GSK-3β/β-catenin signaling pathway.

### LMWCP Increases Hair Inductivity of hDPCs

**Fig. 3:**
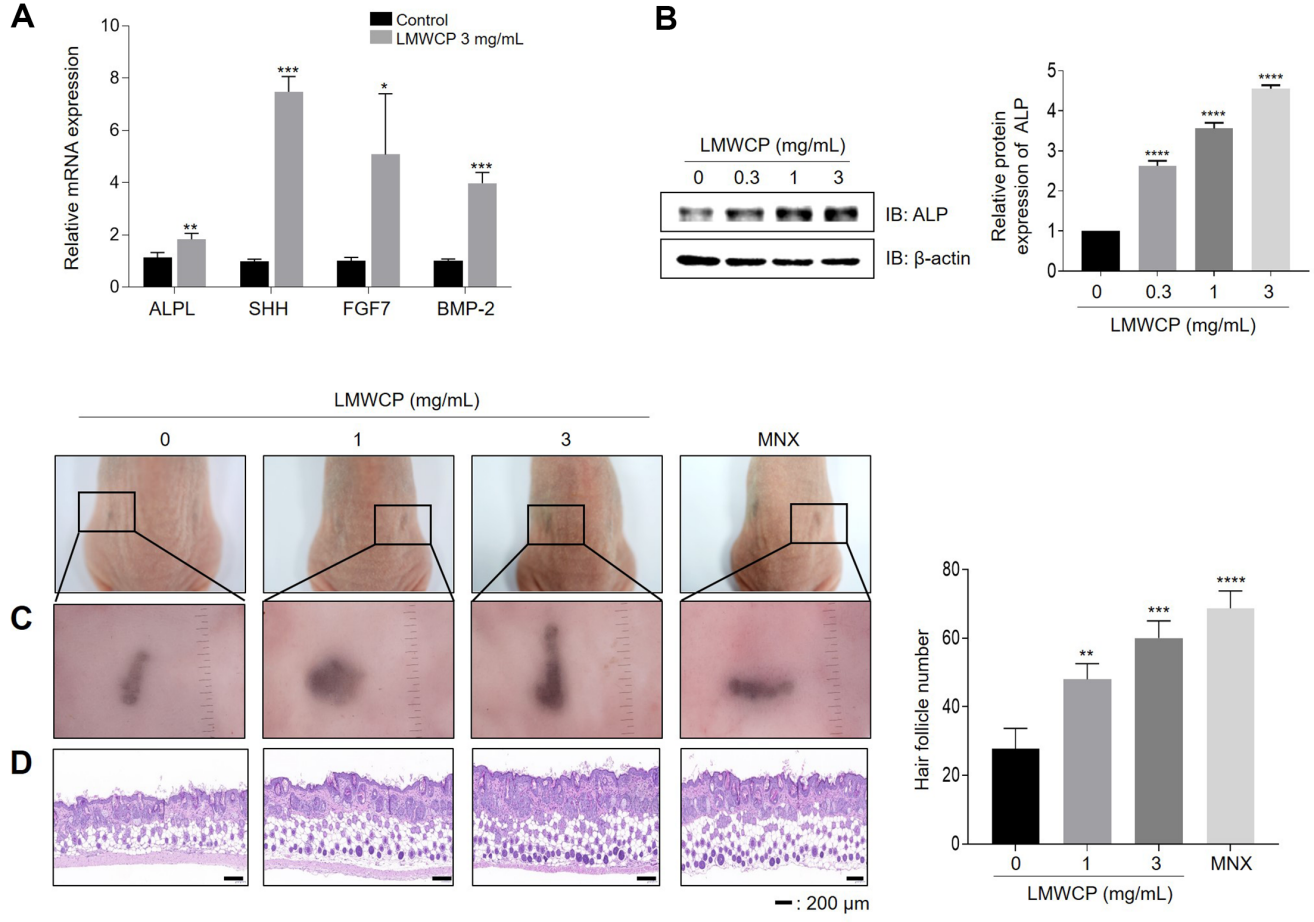
Effect of LMWCP on potential hair inductivity of hDPCs. (**A**) The mRNA expression levels of *ALPL*, *SHH*, *FGF-7*, and *BMP-2* were analyzed by qPCR (*n* = 3). (**B**) hDPCs treated with LMWCP for 24 h were analyzed using western blotting for ALP expression. (**C, D**) Patch assay. At 2 weeks, nude mice were euthanized, and newly generated hair follicles on the back skin were counted using H&E staining. Scale bar, 200 μm. Bar graph shows the number of hHFs in back skin. Results are presented as the mean ± SD of data from three independent experiments. *, *p* < 0.05; **, *p* < 0.01; ***, *p* < 0.001; ****, *p* < 0.0001 compared to the control group.

The tendency of DPCs to aggregate is associated with inductivity of hair growth [26, 27]. We evaluated the effect of LMWCP on hair inductivity using a three-dimensional (3D) spheroid culture. LMWCP promoted the aggregation of hDPCs spheres after 2 days compared to the control (Fig. S1). Next, we investigated the expression levels of DP signature genes after LMWCP treatment using qRT-PCR. The expression levels of *ALPL*, Sonic hedgehog (*SHH*), fibroblast growth factor-7 (*FGF-7*), and bone morphogenetic protein-2 (*BMP-2*) were significantly elevated in LMWCP-treated hDPCs compared with the control group (Fig. 3A). Particularly, the expression of ALP, which improves the hair inductivity of hDPCs [28], was upregulated in a dose dependent manner after LMWCP treatment (Fig. 3B). To confirm the effect of LMWCP on new hair inductivity in *ex vivo*, we conducted a patch assay. LMWCP-treated mixed epidermis and dermis cells exhibited promoted new HFs induction compared to the vehicle-treated group (Fig. 3C and 3D).

### LMWCP Enhances hHF Growth in an *ex vivo* Model

**Fig. 4:**
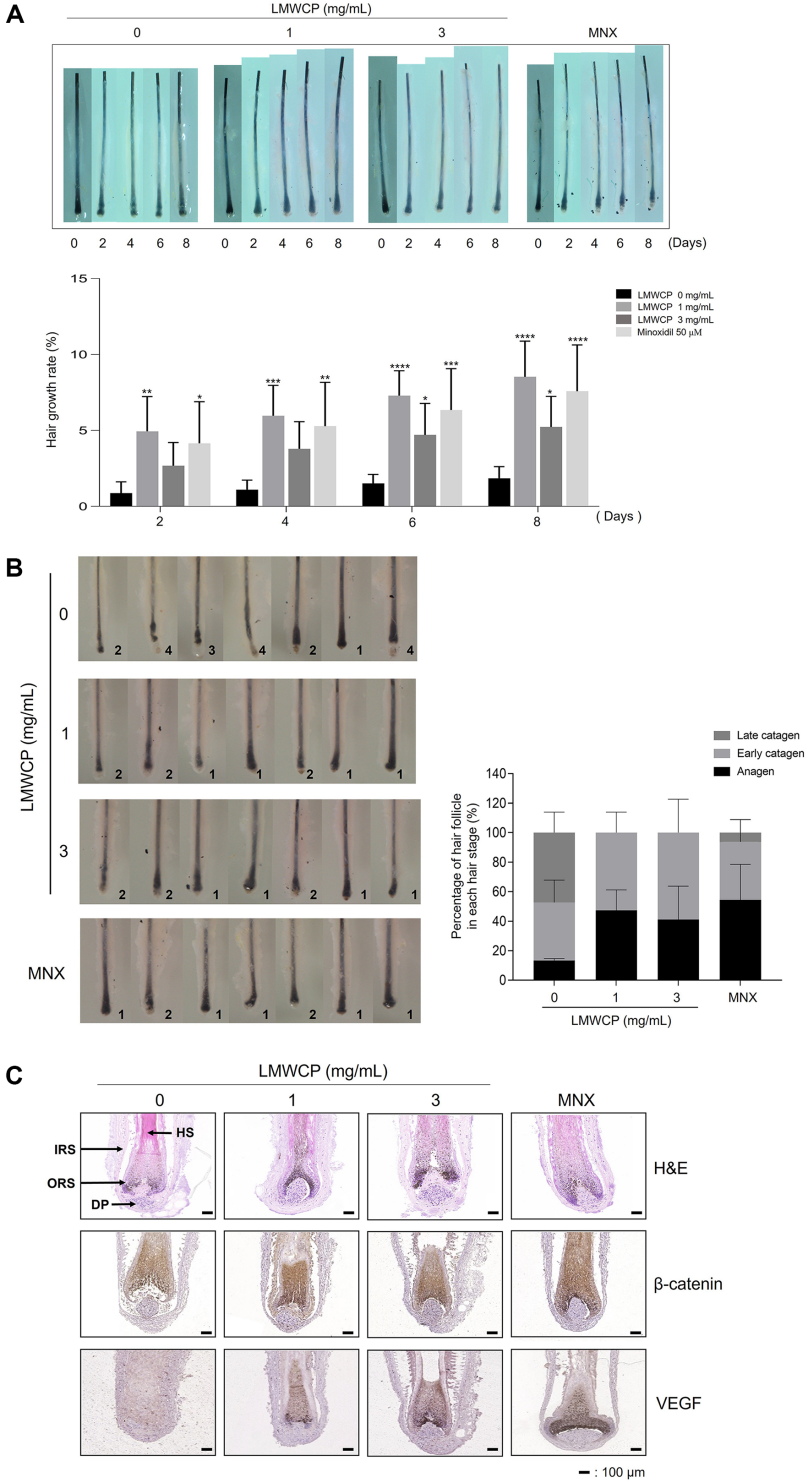
Effect of LMWCP on hair elongation in a hHF organ culture model. The hHFs (8 hair follicles/group) were treated with LMWCP (0, 1, and 3 mg/ml) or MNX (50 μM) for 8 days. (**A**) HFs length was analyzed under a stereomicroscope on days 0, 2, 4, 6, and 8. The relative length of each hair shaft was measured using the ImageJ software. (**B**) After 10days of culture, the HFs phase was assessed following the hair cycle scoring criteria. Representative images of the HFs for each experimental group are shown, as well as the calculated ratios of the hair cycle phases. (**C**) H&E staining and IHC staining of β- catenin and VEGF. The results are shown as the mean ± standard deviation. *, *p* < 0.05; **, *p* < 0.01; ***, *p* < 0.001; ****, *p* < 0.0001 compared to the control group.

HFs are organs containing DPCs. hHFs treated with LMWCP (1 and 3 mg/ml) exhibited longer growth compared to the control hHFs at day 8, similar to the hHFs treated with MNX (50 μM) (Fig. 4A). After treatment with LMWCP, hair cycle of hHFs in 8 days was analyzed using cycle scoring criteria of the HFs (Fig. S2). LMWCP increased the number of hHFs in anagen stage, indicating that LMWCP prolonged the anagen phage of hair cycle in hHFs compared to the vehicle control (Fig. 4B). In addition, IHC staining showed that LMWCP increased the expression levels of β-catenin and VEGF in hHFs compared with the vehicle control (Fig. 4C).

### LMWCP Accelerates Hair Growth in Telogenic C57BL/6 Mice

**Fig. 5:**
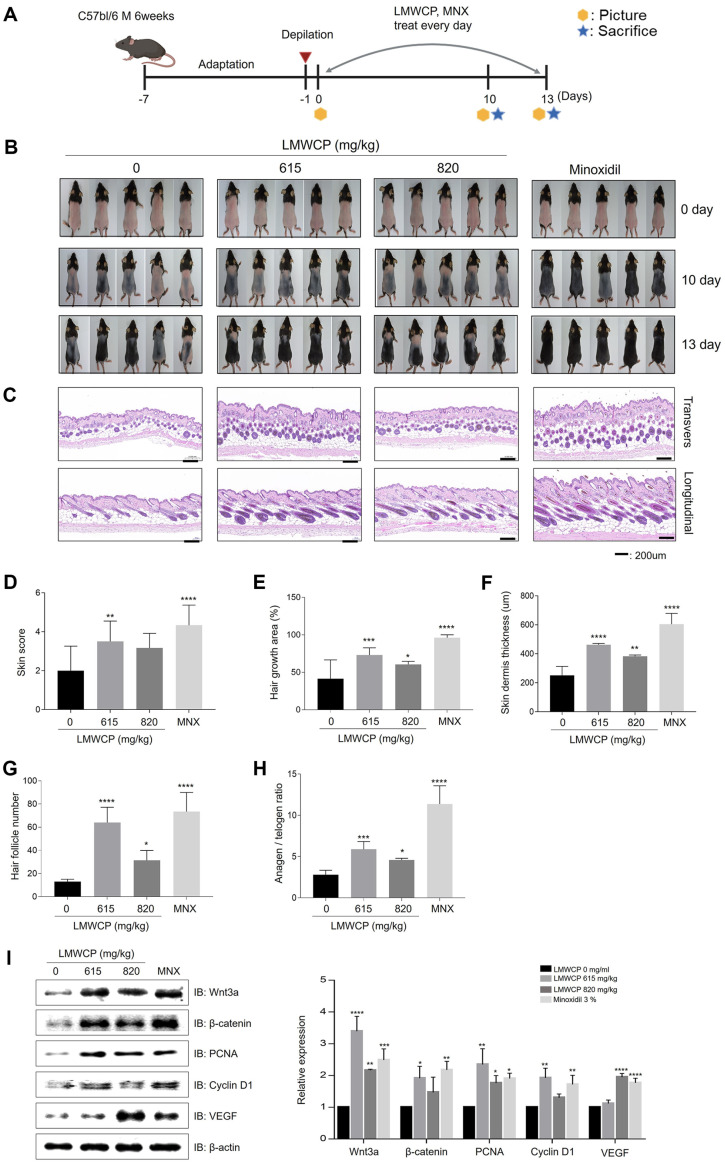
Effect of the LMWCP on anagen induction in 7-week-old female C57BL/6 mice. (**A**) Timetable of experimental treatments and sample collection. (**B**) Representative photographs of mouse back skin on days 0, 10, and 13. (**C**) Representative images of H&E-stained longitudinal and transverse sections of the skin of each mouse on day 13. Scale bar, 200 μm. (**D**) Skin color scores for 10 days. (**E**) Hair growth area on the back skin observed for 13 days. (**F**) Hair dermis thickness, (**G**) HF number, and (**H**) anagen/telogen ratios on day 13. (**I**) The expression levels of Wnt3a, β-catenin, PCNA, cyclin D1, and VEGF on the dorsal skin at day 13. The results are expressed as the mean ± standard deviation. *, *p* < 0.05; **, *p* < 0.01; ***, *p* < 0.001; ****, *p* < 0.0001 compared to the control group.

Depilated mice (7 weeks old) were orally injected with either the vehicle (saline) or LMWCP (615 and 820 mg/kg, referred to as LMWCP 615 and LMWCP 820, respectively) every day for 13 days (Fig. 5A). Mouse skin color score index measurements (Fig. S3) on day 10 showed the highest score in the order of MNX, LMWCP 615, and LMWCP 820 groups (Fig. 5B and 5D). After 13 days, the area of hair regrowth was significantly higher (*p*<0.001) in the LMWCP 615 and LMWCP 820 groups, along with the MNX group, compared to the negative control group (Fig. 5B and 5E). The thickness of the interfollicular whole skin also significantly increased in LMWCP-treated and MNX-treated mice (*p*<0.05) (Fig. 5C and 5F). Analysis of representative longitudinal (HF number) and transverse (hair growth phase) sections confirmed that the number of total HFs and anagen HFs were elevated in the LMWCP‐treated groups compared to the vehicle group (Fig. 5C, 5G, and 5H). The increases in the protein levels of Wnt3a, β-catenin, PCNA, cyclin D1, and VEGF were confirmed through western blot analysis (Fig. 5I). Furthermore, we confirmed that β-catenin and VEGF significantly increased in the back skin of the mice using IHC (Fig. S4). Collectively, these results indicate that LMWCP promotes hair growth by stimulating the transition from telogen phase to anagen phase.

### LMWCP Increases the Expression of Keratins

**Fig. 6:**
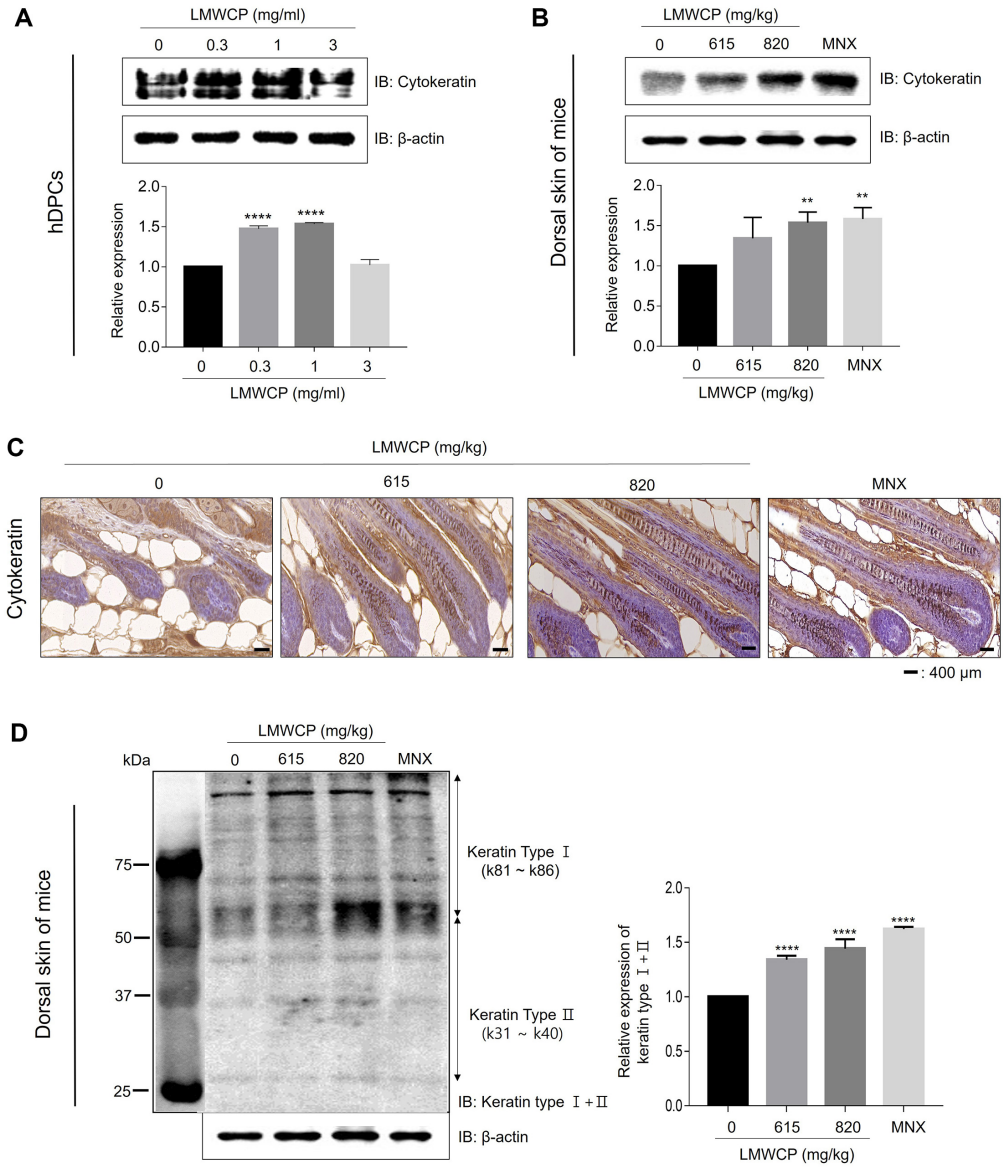
LMWCP increases the expression of keratin in hDPCs and the dorsal skin of mice. (**A**) Western blotting analysis showing the total cytokeratin levels in hDPCs treated with 0, 0.3, 1, and 3 mg/ml of LMWCP for 24 h. (**B–D**) Western blotting and IHC analysis showing the total cytokeratin and Type I + II hair keratin levels in the dorsal skin of the exposed LMWCP (615 and 820 mg/kg) and MNX treatment and control mice after 13 days. Representative western blotting and IHC images from four independent experiments are shown. β-actin was used as a loading control. Scale bar, 400 μm. The results are expressed as the mean ± standard deviation. **, *p* < 0.01; ****, *p* < 0.0001 compared to the control group.

Keratin is a primary component of hair. Throughout the keratinization process, numerous keratins organize into protein filaments to participate in the assembly of the hair shaft within follicle bulbs [29]. We investigated whether a LMWCP affects the expression of keratin in hDPCs and back skin of mice. The cytokeratin total levels were evaluated using a broad-spectrum anti-pan-cytokeratin antibody. LMWCP significantly increased the cytokeratin levels in those cells and tissues compared to the controls (Fig. 6A and 6B). As shown in Fig. 6C, following the LMWCP treatment, it was confirmed that the expression of cytokeratins increased throughout the hair follicle. Next, to explore the biological effects of the LMWCP on the hair keratin expression, both Type I and II hair keratins were determined by Western blot analysis. The expression of Type I and II hair keratins accelerated in the dorsal skin administrated with the LMWCP (Fig. 6D). Overall, these results suggest that the LMWCP can promote hair growth by increasing keratin production.

## Discussion

Currently, there is growing interest in hair loss prevention and hair health. LMWCP has received special attention as it is an essential component of skin and hair, and it is considered a safe natural ingredient.

Mitochondria play an important role in follicle regeneration, as mitochondrial aerobic respiration is activated during hair follicle stem cell differentiation, and its dysfunction retards hair regeneration [24, 25]. Moreover, the stimulation of mitochondrial function prolongs anagen phase, enhances hair follicle keratinocyte proliferation, and modifies intra-follicular keratin expression [29-31], indicating that mitochondrial activity play an essential role in maintaining hair growth. In the present study, we observed that LMWCP increased not only hDPCs proliferation but also mitochondrial potential (Fig. 1A and 1C). These findings suggest that the increase in hDPCs proliferation in response to LMWCP treatment may be associated with activating mitochondrial potential.

hDPCs secrete several growth factors on the HFs to promote hHFs’ growth [9]. Any changes in the distribution of the relevant growth factor receptors and their expression levels can cause abnormalities in the growth and development of hair follicles [32, 33]. As shown in Fig. 1F, we found that LMWCP stimulated the secretion of EGF. The EGF interacts with the epidermal growth factor receptor (EGFR) in the outer root sheath (ORS) of mature hHFs, which induces DNA synthesis in ORS cells and differentiates hair bulb cells into ORS cells. In addition, EGF plays an inhibitory role in hair follicle formation during the initial stages of hair follicle growth [34]. Using *ex vivo* and in vivo models (Figs. 4A and 5B), we found that LMWCP increased the growth of both hHFs and mouse hair. These growth-stimulating effects may be mediated with the increased secretion of growth factors acting on the hHFs.

Next, we observed that LMWCP promoted the aggregation of hDPCs (Fig. S1) and upregulated the expression of DP signature genes such as *ALPL*, *SHH*, *FGF7*, and *BMP-2* (Fig. 3A). Hyunju *et al*. reported that collagne13A1 (Col13A1) and collagne15A1 (Col15A1) induce the spheroid formation of hDPCs. This collagen expression is downregulated in aged HFs, and aged hDPCs are difficult to aggregate. Blocking COL13A1 and COL15A1 expression using small interfering RNA has been found to reduce aggregation and induce senescence of hDPCs in vitro [18]. These findings indicate that collagen plays pivotal roles in spheroid formation and support our finding that LMWCP induces hair growth by increasing hDPCs’ hair inductivity.

Cyclic hair growth depends on the induction of angiogenesis to meet the increased nutritional needs of hair follicles during the anagen phase of rapid cell division [35, 36]. VEGF, as an autocrine growth factor for hDPCs, can stimulate the proliferation and migration of hDPCs [37]. Interestingly, LMWCP induces a dose-dependent increase in VEGF expression in hDPCs (Fig. 2B). In addition, VEGF expression was upregulated in human HFs and mouse hair shafts in response to LMWCP treatment (Figs. 4C, 5I, and S4). These results suggest that LMWCP could promote the supplement of nutrients by increasing angiogenesis during hair growth.

Hair growth could be regulated by modulating the hair cycle, for example by prolonging the anagen phase or promoting the telogen-to-anagen transition [38, 39]. C57BL/6 mice possess melanocytes only in the hair follicles. and melanin synthesis occurs with the hair growth cycle [40]. Thus, change of the hair growth cycle can be easily identified by simply monitoring the transition of the skin color from pink (no hair) to black (fully grown hair)[41]. As shown in Fig. 5B and 5D, the skin score was higher in mice receiving 820 mg/kg LMWCP, indicating that LMWCP induces the telogen-anagen transition earlier. The results demonstrated that LMWCP increases hair growth by stimulating telogen transition of hair cycle.

Hair keratins constitute up to 95% of the hair structure and contribute to the mechanical strength of the cells [42]. Recently, Seong *et al*. found that keratin is critical for condensation of hDPCs and generation of a P-cadherin-expressing cell population (hair germ) from outer root sheath cells [43], indicating that keratin can promote the hair growth by increasing the hair inductivity of hDPCs. In our study, LMWCP increased the expression of cytosolic keratin in hDPCs and the dorsal skin of mice (Fig. 6A-6C). In addition, the expression levels of hair keratin type I and type II were increased in the dorsal skin of LMWCP-treated mice (Fig. 6D). These results indicate that LMWCP could play a crucial role in increasing hair growth by increasing the expression of keratin.

## Conclusion

The results of the present study provide the first evidence that LMWCP contributes to the growth and cycling of hair through the Wnt-AKT-GSK-3β/β-catenin signaling pathway. LMWCP enhances new hair formation by increasing the secretion of growth factors and promoting hair inductivity. Moreover, we observed that oral administration of LMWCP during the telogen phase accelerated the onset of the anagen phase and increased the expression of VEGF and β-catenin. Collectively, LMWCP can be used as a supplement to alleviate the symptoms of hair loss.

## Supplemental Materials

Supplementary data for this paper are available on-line only at http://jmb.or.kr.



## Figures and Tables

**Table 1:** Primer sequences used for quantification of gene expression.

Gene		Primer sequence (5'→ 3')
Human EGF	F	CAGGGAAGATGACCACCACT
	R	CAGTTCCCACCACTTCAGGT
Human HB-EGF	F	ACAAGGAGGAGCACGGGAAAAG
	R	CGATGACCAGCAGACAGACAGATG
Human FGF-4	F	GGGAGTCTACAGACAGCAAG
	R	GAGCCTAGGGTGTGGTTTA
Human FGF-6	F	GGGAGTCTACAGACAGCAAG
	R	GAGCCTAGGGTGTGGTTTA
Human ALPL	F	ATTGACCACGGGCACCAT
	R	CTCCACCGCCTCATGCA
Human SHH	F	GCGCCAGCGGAAGGTAT
	R	CCGGTGTTTTCTTCATCCTTAAA
Human FGF7	F	ATCAGGACAGTGGCAGTTGGA
	R	AACATTTCCCCTCCGTTGTGT
Human BMP-2	F	GAGGTCCTGAGCGAGTTCGA
	R	TCTCTGTTTCAGGCCGAACA

**Table 2:** Antibodies used for Western blot analysis.

Antibodies	Product code	Company
Anti-MITF	MAB3747-I	Sigma-Aldrich (MO, USA)
Anti-Calnexin	ab22595	Abcam (Cambridge, UK)
Anti-ALIX	ab275377	Abcam (Cambridge, UK)
Anti-CD63	ab134045	Abcam (Cambridge, UK)
Anti-tyrosinase	ab180753	Abcam (Cambridge, UK)
Anti-p-MITF	ab59201	Abcam (Cambridge, UK)
Anti-TRP-1	sc-58437	Santa Cruz Biotechnology (CA, USA)
Anti-TRP-2	sc-25544	Santa Cruz Biotechnology (CA, USA)
Anti-Rab27a	sc-22756	Santa Cruz Biotechnology (CA, USA)
Anti-β-actin	sc-47778	Santa Cruz Biotechnology (CA, USA)
Anti-p-CREB	#9198	Cell Signaling Technology Inc. (Beverly, MA)
Anti-CREB	#9197	Cell Signaling Technology Inc. (Beverly, MA)
Anti-p-ERK	#9101	Cell Signaling Technology Inc. (Beverly, MA)
Anti-ERK	#9102	Cell Signaling Technology Inc. (Beverly, MA)
Anti-p-AKT	#4060	Cell Signaling Technology Inc. (Beverly, MA)
Anti-AKT	#4691	Cell Signaling Technology Inc. (Beverly, MA)
Anti-p-β-catenin	#4176	Cell Signaling Technology Inc. (Beverly, MA)
Anti-β-catenin	#8480	Cell Signaling Technology Inc. (Beverly, MA)
Anti-Myosin-Va	#3402	Cell Signaling Technology Inc. (Beverly, MA)
Anti-MLPH	10338-1-AP	ProteinTech Group (IL, USA)
