# Corrigendum to: Low Molecular Weight Collagen Peptide (LMWCP) Promotes Hair Growth by Activating the Wnt/GSK-3β/β-Catenin Signaling Pathway

**DOI:** 10.4014/jmb.2024.3404.985

**Published:** 2024-04-28

**Authors:** Yujin Kim, Jung Ok Lee, Jung Min Lee, Mun-Hoe Lee, Hyeong-Min Kim, Hee-Chul Chung, Do-Un Kim, Jin-Hee Lee, Beom Joon Kim

**Affiliations:** 1 Department of Dermatology, College of Medicine, Chung-Ang University, Seoul 06974, Republic of Korea; 2 Department of Medicine, Graduate School, Chung-Ang University, Seoul 06973, Republic of Korea; 3 Health Food Research and Development, NEWTREE Co., Ltd., Seoul 05604, Republic of Korea

In the article titled “**Low Molecular Weight Collagen Peptide (LMWCP) Promotes Hair Growth by Activating the Wnt/GSK-3β/β-Catenin Signaling Pathway**”, the authors noticed that the table included in this paper, which pertains to antibodies used in other whitening studies besides those actually employed in this study. We will provide the accurately revised table with only the correct antibody information included.

In the article, we used table2. Antibodies used for Western blot analysis.

**Table:** 

Antibodies	Product code	Company
Anti-MITF	MAB3747-I	Sigma-Aldrich (MO, USA)
Anti-Calnexin	ab22595	Abcam (Cambridge, UK)
Anti-ALIX	ab275377	Abcam (Cambridge, UK)
Anti-CD63	ab134045	Abcam (Cambridge, UK)
Anti-tyrosinase	ab180753	Abcam (Cambridge, UK)
Anti-p-MITF	ab59201	Abcam (Cambridge, UK)
Anti-TRP-1	sc-58437	Santa Cruz Biotechnology (CA, USA)
Anti-TRP-2	sc-25544	Santa Cruz Biotechnology (CA, USA)
Anti-Rab27a	sc-22756	Santa Cruz Biotechnology (CA, USA)
Anti-β-actin	sc-47778	Santa Cruz Biotechnology (CA, USA)
Anti-p-CREB	#9198	Cell Signaling Technology Inc. (Beverly, MA)
Anti-CREB	#9197	Cell Signaling Technology Inc. (Beverly, MA)
Anti-p-ERK	#9101	Cell Signaling Technology Inc. (Beverly, MA)
Anti-ERK	#9102	Cell Signaling Technology Inc. (Beverly, MA)
Anti-p-AKT	#4060	Cell Signaling Technology Inc. (Beverly, MA)
Anti-AKT	#4691	Cell Signaling Technology Inc. (Beverly, MA)
Anti-p-β-catenin	#4176	Cell Signaling Technology Inc. (Beverly, MA)
Anti-β-catenin	#8480	Cell Signaling Technology Inc. (Beverly, MA)
Anti-Myosin-Va	#3402	Cell Signaling Technology Inc. (Beverly, MA)
Anti-MLPH	10338-1-AP	ProteinTech Group (IL, USA)

Actually, we used the antibodies as below.

**Table 2:** Antibodies used for Western blot analysis.

Antibodies	Product code	Company
Anti-PCNA	#2586	Cell Signaling Technology Inc. (MA, USA)
Anti-Cyclin D1	#2978	Cell Signaling Technology Inc. (MA, USA)
Anti-CDK2	#2546	Cell Signaling Technology Inc. (MA, USA)
Anti-p-AKT (Ser473)	#4060	Cell Signaling Technology Inc. (MA, USA)
Anti-AKT	#4691	Cell Signaling Technology Inc. (MA, USA)
Anti-p-GSK-3β (Ser9)	#93365	Cell Signaling Technology Inc. (MA, USA)
Anti-total GSK-3β	#12456	Cell Signaling Technology Inc. (MA, USA)
Anti-p-β-catenin (Ser675)	#4176	Cell Signaling Technology Inc. (MA, USA)
Anti-total β-catenin	#8480	Cell Signaling Technology Inc. (MA, USA)
Anti-LEF	#2230	Cell Signaling Technology Inc. (MA, USA)
Anti-VEGF	#50661	Cell Signaling Technology Inc. (MA, USA)
Anti-β-actin	#3700	Cell Signaling Technology Inc. (MA, USA)
Anti-Cyclin E	ab33911	Abcam (Cambridge, UK)
Anti-CDK6	ab124821	Abcam (Cambridge, UK)
Anti-ALP	PA5-106391	Thermo Fisher Scientific (MA, USA)
Anti-Cytokeratin	C5992	Sigma-Aldrich (MO, USA)
Anti- type I+II hair keratin	GP-PANHK	PROGEN Biotechnical GmbH (Heidelberg Germany)
Horse Anti-Mouse IgG	PI-2000-1	Vector Laboratories (CA, USA)
Goat Anti-Rabbit IgG	PI-1000-1	Vector Laboratories (CA, USA)
anti-rabbit IgG-FITC	sc-2359	Santa Cruz Biotechnology (TE, USA)

