# Supplementary material for: Low Molecular Weight Collagen Peptide (LMWCP) Promotes Hair Growth by Activating the Wnt/GSK-3β/β-Catenin Signaling Pathway
Source: J Microbiol Biotechnol. 2023 Sep 30;34(1):17–28. doi: 10.4014/jmb.2308.08013 (PMC10840484; doi:10.4014/jmb.2308.08013)
Supplement: Supplementary file 1 [file jmb-34-1-17-supple.pdf]

Supplementary Figures Tables

Low molecular weight collagen peptide (LMWCP) increases hair growth via activation of the GSK-3β/β-catenin signaling pathway

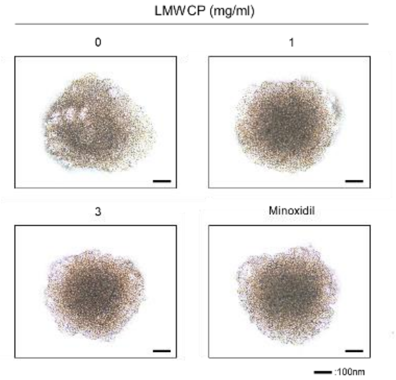

Figure S1. The effect of LMWCP on hair inductivity analyzed using a three-dimensional (3D) spheroid culture. Related to Figure 3. Comparison of spheroid formation in the control, LMWCP-treated (1 or 3 mg/mL), and MNX-treated hDPC cells. Images were analyzed 2 days after LMWCP treatment.

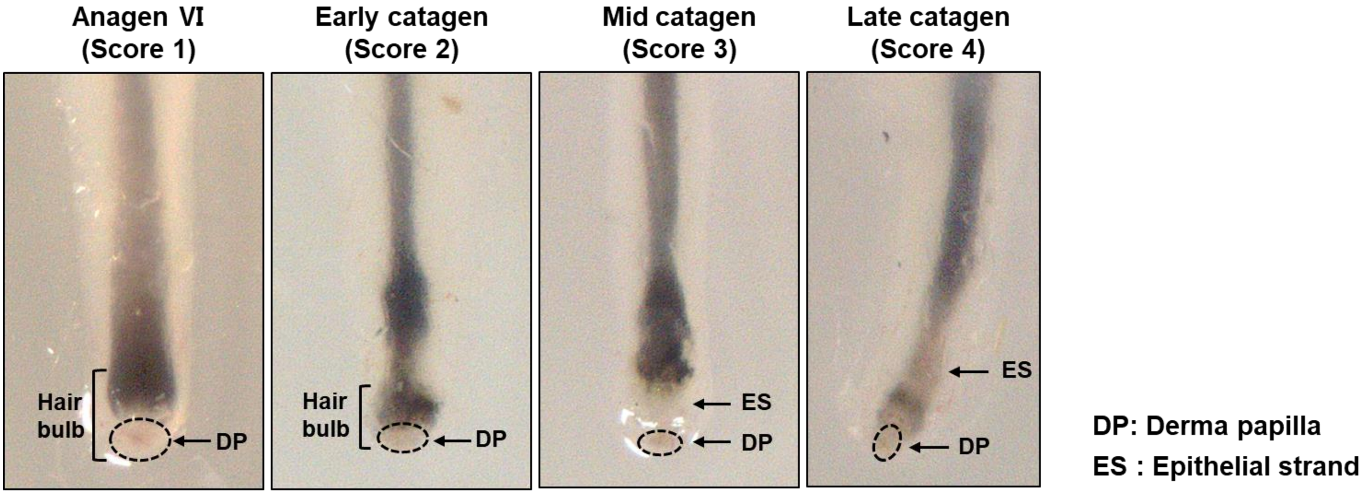

Figure S2. Cycle scoring criteria of the hair shaft from anagen to catagen. Related to figure 4. The standard image shows the hair cycle scoring criteria of the hair shaft, including anagen VI (score 1), early catagen (score 2), mid-catagen (score 3), and late catagen (score 4). During anagen VI (score 1), the size of the dermal papilla (DP) in the hair shaft increases, and the hair bulb surrounding the DP also enlarges. At early catagen (score 2), the DP takes on an almond-shape and condensed appearance, while the volume of the hair bulb decreases. During mid-catagen (score 3), the epithelial strand (ES) appears without pigmentation. At late catagen, (score 4) the ES is longer, and the DP forms a ball-shaped structure.

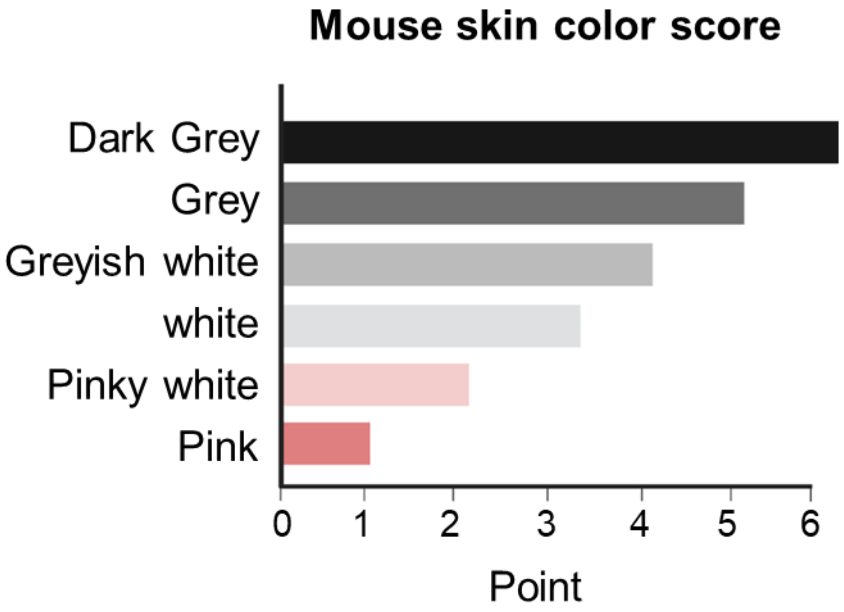

Figure S3. The skin color score of the C57BL/6 mice. Related to figure 5. A score of 1 indicates pink skin with no hair growth; 2, pinky white skin; 3, white skin indicating early anagen; 4, greyish white skin and hair growth lesion is less than 30%; 5, grey skin and hair growth lesion is 30–70%; 6, dark grey skin and hair growth lesion is over 90%.

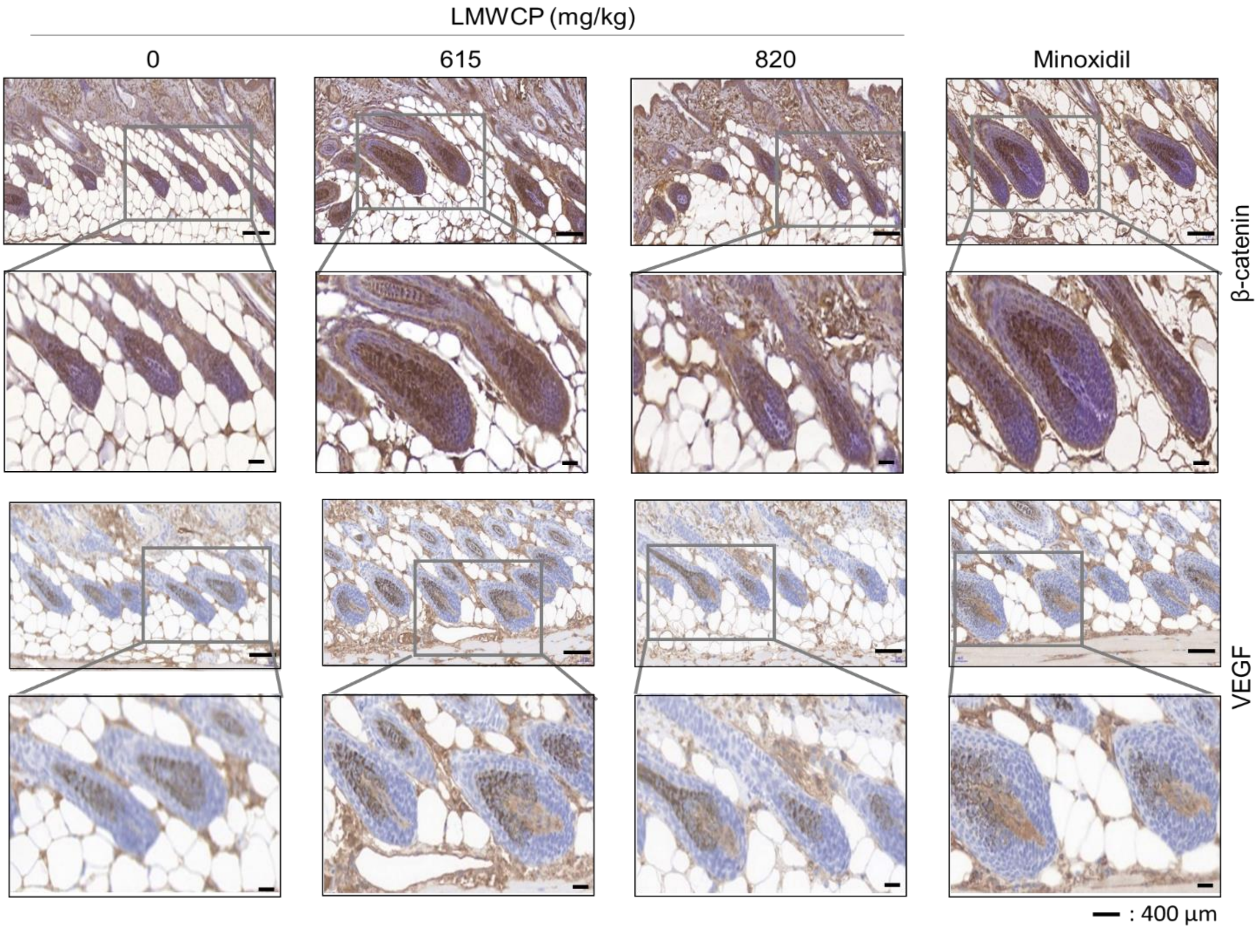

Figure S4. Effect of LMWCP on the Wnt/β-catenin signaling pathway. Related to Figure 5. The expression levels of β-catenin and VEGF on the dorsal skin at day 13. The results are expressed as the mean ± standard deviation. *, $p < 0.05$; **, $p < 0.01$; ***, $p < 0.001$; ****, $p < 0.0001$ compared with the control group. LMWCP, low molecular weight collagen peptide.
